# The function of mRNA quality control in aging and age-related diseases

**DOI:** 10.1016/j.jbc.2026.111351

**Published:** 2026-03-06

**Authors:** Seokjun G. Ha, Hyunwoo C. Kwon, Jongsun Lee, Hyung-Jun Kim, Seung-Jae V. Lee

**Affiliations:** 1 Department of Biological Sciences, Korea Advanced Institute of Science and Technology, Daejeon, South Korea; 2 Dementia Research Group, Korea Brain Research Institute (KBRI), Daegu, South Korea

**Keywords:** aging, mRNA quality, age-related diseases, NMD, RQC

## Abstract

Aging is a complex biological process characterized by the gradual decline of physiological and molecular functions and increased susceptibility to age-associated diseases. Emerging evidence indicates the role of mRNA quality control mechanisms in the regulation of aging and longevity. This review focuses on the function of mRNA surveillance mechanisms, including nonsense-mediated mRNA decay, nonstop decay, and no-go decay, in aging and age-related diseases. We discuss the critical roles of these pathways in maintaining mRNA quality and preventing the accumulation of aberrant transcripts, which can contribute to aging and age-related disorders. Specifically, we discuss the function of nonsense-mediated mRNA decay in aging processes and age-related diseases, including cancer and neurodegenerative disorders. We also review the safeguarding roles of nonstop decay and no-go decay in preventing the accumulation of faulty mRNAs and proteins associated with various diseases. We explore the potential functions of additional mRNA surveillance and the associated signaling pathways, such as ribosome-associated quality control, in aging and age-related diseases. Understanding the intricate relationship between mRNA surveillance mechanisms and aging may provide key information for developing potential therapeutics that boost these pathways for delaying aging and treating age-related diseases.

Aging is accompanied by a gradual decline in physiological functions and an exponential increase in susceptibility to multiple age–associated diseases. Aging is caused by the impairment of biological systems at multiple levels. At the cellular level, the accumulation of senescent cells, which stably stop proliferation, is considered a major cause of aging (1). At the molecular level, genomic instability and reduced proteostasis contribute to accelerating both cellular senescence and organismal aging (2, 3). Recent studies also suggest important roles of mRNA quality control systems in aging. Studies using the nematode *Caenorhabditis elegans* demonstrated the important function of mRNA quality control and homeostatic regulation of splicing in organismal aging (4, 5, 6, 7, 8, 9). In addition, age-dependent accumulation of stalled ribosomes, which are closely associated with cotranslational mRNA quality control systems, contributes to aging and longevity in the budding yeast *Saccharomyces cerevisiae* and *C*. *elegans* (10, 11).

Eukaryotic cells are equipped with multiple mRNA surveillance systems that eliminate abnormal transcripts. Nonsense-mediated mRNA decay (NMD), a key RNA surveillance process, targets mRNA transcripts that contain premature termination codons (PTCs). Nonstop decay (NSD) eliminates mRNAs without stop codons that cause ribosome stalling at the poly(A) tail, and conventionally, no-go decay (NGD) removes mRNAs with internal stem–loop structures or rare codons that cause internal ribosome stalling. Although poly(A)-mediated ribosome stalling has been classically associated with NSD, recent studies showed that poly(A) stretches can trigger ribosome collisions and activate NGD, indicating a partial mechanistic overlap between the two pathways (12, 13, 14, 15). Slow elongation caused by nonoptimal or rare codons activates a noncanonical mRNA surveillance pathway, codon-optimality–mediated decay, rather than NGD, and the decay of such mRNAs is mechanistically distinct from NGD (16). Impairments of NMD, NSD, and NGD contribute to physiological defects, such as premature aging and neurodegeneration (9, 17, 18), highlighting the importance of proper maintenance of mRNA quality control in organismal health. Here, we discuss the current status of our understanding regarding the role of mRNA surveillance mechanisms in the prevention of aging and various age-related diseases.

## Basic concepts of mRNA surveillance mechanisms

**Figure 1:**
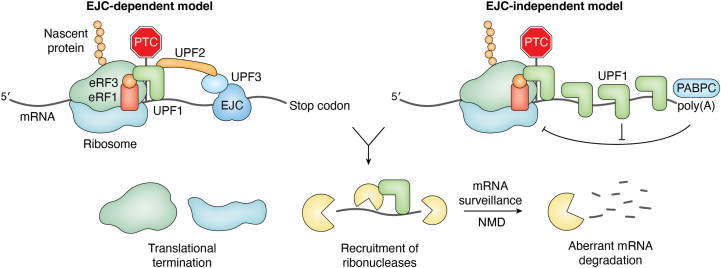
**Schematic of mRNA surveillance by nonsense-mediated mRNA decay (NMD)**. NMD eliminates abnormal transcripts with premature termination codons (PTCs). In the exon–junction complex (EJC)–dependent model, NMD is initiated by a PTC that is located upstream of the binding sites of EJCs. Suppressor with morphogenetic effect on genitalia-2/up-frameshift 1 (SMG-2/UPF1) and SMG-4/UPF3 recognize eukaryotic release factors 1 and 3 (eRF1 and eRF3) by binding to eRF3 of PTC-encountered ribosomes and the EJC, respectively. SMG-3/UPF2 bridges SMG-2/UPF1 and SMG-4/UPF3, activating NMD. In the EJC-independent model, poly(A)-binding protein cytoplasmic 1 (PABPC1) inhibits the binding of SMG-2/UPF1 to eRF3, and a long distance between a PTC and PABPC1 weakens the binding to eRF3. SMG-2/UPF1 eventually binds to eRF3 and initiates NMD. Ribosomes are then dissociated by translational termination, and SMG-2/UPF1 recruits ribonucleases that degrade aberrant mRNAs for mRNA surveillance.

NMD is the most extensively characterized mRNA surveillance process, which eliminates transcripts with PTCs located upstream of exon–exon junctions. Key factors of NMD include suppressor with morphogenetic effect on genitalia-2/up-frameshift 1 (SMG-2/UPF1), SMG-3/UPF2, SMG-4/UPF3, and SMG-1/SMG1, most of which are conserved from *S*. *cerevisiae* and *C*. *elegans* to humans (19, 20, 21, 22, 23, 24). SMG-2/UPF1 and SMG-1/SMG1 bind to eukaryotic translation termination factor (eRF) 3, which interacts with eRF1 that recognizes stop codons (25). If exon junction complexes (EJCs) exist downstream of stop codons with sufficient distance (>50 nucleotides) (26), SMG-3/UPF2 connects terminating ribosomes and the EJCs (20), forming NMD complexes. After the formation of the NMD complex, SMG-1/SMG1 phosphorylates SMG-2/UPF1, and various components of the mRNA-degrading machinery are recruited (Fig. 1).

Although the classic EJC-dependent NMD model indicates that NMD is initiated by a PTC upstream of an EJC, many studies suggest that there is no absolute rule of NMD. NMD can be triggered in an EJC-independent manner, such as cap-independent translation of mRNAs containing PTCs, translation of intronless mRNAs harboring PTCs, ribosomal frameshifting that generates a PTC, and translation of mRNAs with a long 3′ UTR (27, 28, 29, 30, 31, 32). To explain these, the EJC-independent model of NMD suggests that NMD is initiated by a long distance between poly(A)-binding protein cytoplasmic 1 (PABPC1) and a PTC. PABPC1 antagonizes UPF1-dependent NMD by promoting efficient termination *via* interacting with eRF3 at a PTC, whereas a long distance between a PTC and PABPC1 weakens the terminating interaction and increases UPF1 occupancy, leading to an interaction between eRF3 and UPF1. The interaction between UPF1 and eRF3 eventually leads to NMD. NMD also regulates mRNA levels by targeting transcripts with upstream ORFs (33). Although it remains unclear whether specific gene regulation by NMD is beneficial for organisms, many studies highlight the importance of the maintenance of mRNA quality control by NMD in cellular and physiological functions (6).

**Figure 2:**
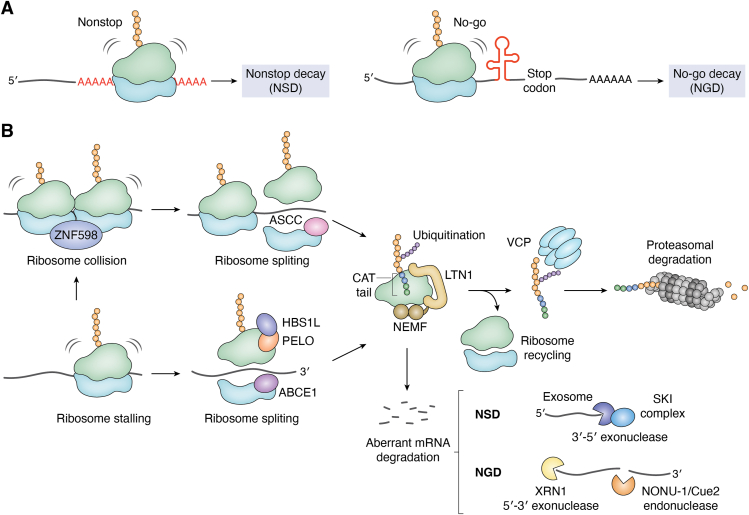
**Schematic of nonstop decay (NSD), no-go decay (NGD), and ribosome-associated quality control (RQC)**. *A*, NSD and NGD act on aberrant mRNAs that cause slow translation, leading to ribosome stalling and collisions. NSD degrades transcripts lacking a stop codon. NGD degrades transcripts containing obstacles such as secondary structures. *B*, collided ribosomes following ribosome stalling are recognized and ubiquitinated by the zinc finger protein 598 (ZNF-598/ZNF598). At the site of collision, the leading ribosome is disassembled by the activating signal cointegrator 1 complex (ASCC). Ribosomes stalled at the 3′ end of an mRNA are rescued by the PELOTA (PELO-1/DOM34/PELO) in conjunction with the HBS1-like translational GTPase (HBS-1/HBS1/HBS1L), which recruits the ATP-binding cassette subfamily E member 1 (ABCE-1/ABCE1) to split the ribosome. Aberrant mRNAs are subsequently degraded by pathway-specific nucleases. In the NSD pathway, the superkiller (SKI) complex with the exosome degrades mRNAs *via* its 3′-5′ exonuclease activity. In the NGD pathway, the endonuclease nonstop nuclease 1/coupling of ubiquitin conjugation to endoplasmic reticulum degradation (NONU-1/Cue2) and the 5′-3′ exoribonuclease 1 (XRN-1/XRN1) degrade the mRNAs. Nascent peptides retained on the 60S subunits are ubiquitinated by the LISTERIN E3 ubiquitin protein ligase 1 (Y54E10A.11/Ltn1/LTN1). The nuclear export mediator factor (Y82E9BR.18/RQC2/Clbn/NEMF) binds to the 60S subunit and promotes carboxy-terminal alanine–threonine (CAT) tail extension. The valosin-containing protein (CDC48/VCP) then extracts the ubiquitinated nascent peptide from the 60S subunit. The released peptide undergoes proteasomal degradation, preventing the accumulation of aberrant proteins, and the remaining ribosomal subunits are recycled.

NSD and NGD, the other two established cotranslational mRNA surveillance processes, target faulty transcripts that are associated with ribosome stalling or collisions. NSD acts on mRNAs lacking a stop codon, whereas NGD targets mRNAs that cause elongation stalls (Fig. 2*A*). Ribosome stalling and collisions on these mRNAs trigger ribosome-associated quality control (RQC). Zinc finger protein 598 recognizes collided ribosomes and mediates the ubiquitination of the 40S ribosome subunits (12, 34). The ubiquitination of the 40S ribosomal subunit is essential for initiating RQC (12, 34). Once a collision is detected, the activating signal cointegrator 1 complex recognizes the collided ribosomes and, through its helicase activity, disassembles the leading ribosome, resulting in the separation of the 60S and 40S subunits (35, 36). Ribosome stalling at the 3′ end of mRNA triggers distinct ribosome rescue mechanisms (37, 38, 39). The heterodimeric complex of PELOTA (PELO-1/DOM34/PELO) and HBS1-like translational GTPase (HBS-1/HBS1/HBS1L) contributes to the rescue of ribosomes stalled at the 3′ end of the mRNA (37, 38, 39). Recruitment of ATP-binding cassette subfamily E member 1 (ABCE-1/ABCE1) follows the sensing of stalled ribosomes by HBS-1/HBS1/HBS1L and PELO-1/DOM34/PELO to split the ribosome into the 60S and 40S subunits (39). Following the ribosome splitting, the aberrant mRNAs that trigger stalling and collisions are degraded *via* two pathways. In the NSD pathway, the superkiller (SKI)–exosome complex degrades the mRNA through its 3′-5′ exonuclease activity (37). In the NGD pathway, 5′-3′ exoribonuclease 1 (XRN-1/XRN1) degrades the mRNA fragments (40). In yeast, the endonuclease coupling of ubiquitin conjugation to endoplasmic reticulum degradation (Cue2) mediates the cleavage of mRNAs during NGD and NSD, and its functional ortholog nonstop nuclease 1 (NONU-1) performs an analogous role in *C*. *elegans* (41, 42, 43). In mammals, the endonuclease NEDD4-binding protein 2 (N4BP2) has been proposed as a homolog of NONU-1/Cue2 based on sequence and small MutS-related domain similarity (41, 42). Although a conserved endonuclease function in NGD has not been directly demonstrated, the depletion of *N4BP2* stabilizes NSD reporter mRNAs, supporting the functional role of N4BP2 in mammalian NSD (44). Concurrently, the nascent peptide that remains associated with the stalled ribosome is ubiquitinated by LISTERIN E3 ubiquitin protein ligase 1 (Y54E10A.11/Ltn1/LTN1) (45). Nuclear export mediator factor (Y82E9BR.18/RQC2/Clbn/NEMF) is a protein characterized by two globular N- and C-terminal domains that are connected by a kinked M domain and binds to the 60S ribosomal subunit (46). Y82E9BR.18/RQC2/Clbn/NEMF promotes Y54E10A.11/Ltn1/LTN1 activity and orchestrates the extension of carboxy-terminal alanine–threonine (CAT) tails, a reaction distinct from canonical translation. Valosin-containing protein (CDC48/VCP) then extracts the Y54E10A.11/Ltn1/LTN1-dependent polyubiquitylated nascent peptide from the 60S ribosomal subunit (46). The released polypeptide is subsequently degraded by the proteasome, thereby preventing the accumulation of aberrant proteins (Fig. 2*B*). The NSD and NGD pathways share several key factors with the RQC pathway (46). The RQC pathway maintains proteostasis by degrading nascent peptides generated from the translation of NSD and NGD target transcripts, which are sources of pathological and abnormal nascent proteins (17, 18).

## The role of NMD in aging and age-related diseases

NMD has recently garnered attention for its potential implications in aging processes. Aging decreases NMD activity in *C*. *elegans*, and proper NMD is required for the longevity of *C*. *elegans* caused by various interventions, including *daf-2*/insulin/IGF-1 receptor mutations (9). Notably, alpha-1,3/1,6-mannosyltransferase (ALGN-2/ALG2), identified as a positive regulator of NMD, contributes to longevity in *C*. *elegans* (5). Consistent with these *C*. *elegans* studies, long promoter-derived transcripts or extended 3′ UTRs, targets of NMD, accumulate in senescent cultured mammalian cells (47). Furthermore, PTC-containing transcripts tend to be upregulated during aging in multiple species, including killifish and mice (48). Premature termination caused by frameshifting, which can be regulated by mRNA decay pathways including NMD, leads to protein misfolding of the conductance regulator chloride channel (cystic fibrosis transmembrane conductance regulator) (32). Although it is not direct evidence of regulation of aging by NMD, this suggests that decreased NMD activity contributes to impaired proteostasis, a possible cause of accelerated aging. Overall, NMD has been identified as a key regulator of aging processes in *C*. *elegans* and mammalian systems.

**Figure 3:**
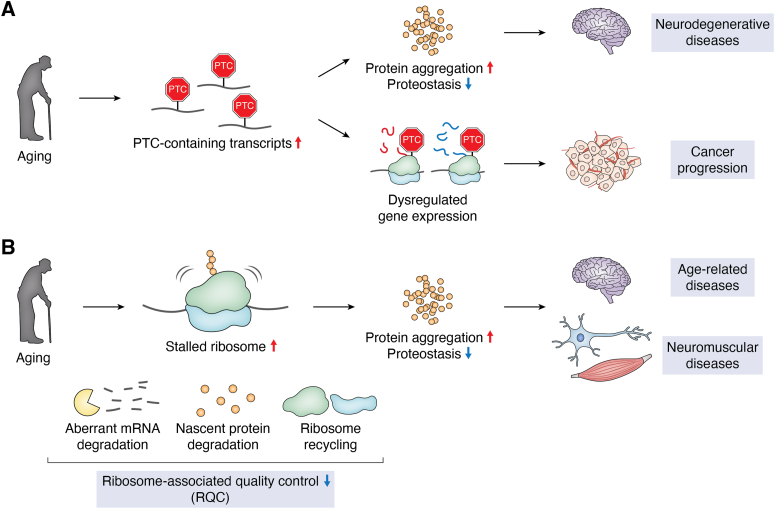
**Regulation of aging and age-related diseases by reduced NMD and RQC**. *A*, during aging, PTC-containing transcripts are upregulated because of reduced NMD activity. Upregulation of PTC-containing transcripts leads to disruption of proteostasis that causes protein aggregation, which is a major cause of neurodegenerative diseases. Genes that are normally repressed by NMD are aberrantly expressed upon the reduction of NMD, leading to cancer progression. *B*, during aging, the level of stalled ribosomes increases. RQC, which resolves stalled ribosomes by promoting aberrant mRNA degradation, nascent protein degradation, and ribosome recycling, becomes less efficient with age. This impaired RQC leads to the accumulation of aberrant and aggregated proteins. These aggregates disrupt proteostasis and contribute to the onset of age-related diseases, including neuromuscular diseases. NMD, nonsense-mediated mRNA decay; PTC, premature termination codon; RQC, ribosome-associated quality control.

NMD plays crucial roles in multiple age–related neurodegenerative diseases (Fig. 3*A*), including Alzheimer's disease (AD), amyotrophic lateral sclerosis (ALS), and Huntington's disease. In *Drosophila melanogaster*, the majority of mRNAs containing differentially retained introns are increased during aging, which potentially activates NMD, and these mRNAs are associated with AD (49). Transgenic expression of human tau genes in a tauopathy model *Drosophila* causes upregulation of NMD targets, approximately by twofold, which is ameliorated by overexpression of *smg-2*/*UPF1* (50). *smg-2*/*UPF1* overexpression elicits neuroprotective functions in various fly and mammalian ALS models (51, 52, 53, 54, 55). NMD regulates the mRNA quality of endoplasmic reticulum–delivered mRNAs, raising the possibility that impairment of NMD may compromise neuronal integrity, leading to neurodegeneration (56). In addition, human SMG-2/UPF1, SMG-3/UPF2, or SMG-4/UPF3 suppresses fused in sarcoma (FUS)–mediated neurotoxicity through the NMD pathway in a yeast ALS model that expresses the human *FUS* gene (57). Loss of NMD also causes a dramatic increase in the level of the short isoform of TAR DNA–binding protein mRNA, whose accumulation causes neurodegeneration (58). These findings suggest important neuroprotective functions of NMD and the therapeutic potential of targeting NMD to treat neurodegenerative diseases.

The pathogenesis of cancer is associated with NMD (Fig. 3*A*). Cancer genomes usually contain many somatic mutations, a subset of which generate PTCs, implicating the role of NMD in the regulation of pivotal cancer-associated genes (59, 60, 61). PTC-containing transcripts encoded by tumor suppressor genes, including *p53* and *p21*, are degraded by NMD (60, 62, 63, 64). The expression of *smg-2*/*UPF1* is significantly decreased in hepatocellular carcinoma (HCC) tissues of 31 of 50 patients, suggesting a general decrease in NMD activity (65). In HCC tissues, the mRNA level of an NMD target, suppressor of mothers against decapentaplegic 7 (*SMAD7*), encoding a transforming growth factor-beta inhibitor, is substantially increased (65, 66). *smg-2*/*UPF1* overexpression reduces the levels of both *SMAD7* mRNA and SMAD7 protein in HCC cells (65). Thus, SMG-2/UPF1 suppresses HCC tumorigenesis by activating the transforming growth factor-beta pathway through downregulation of *SMAD7*. Downregulation of *smg-2*/*UPF1* increases immune evasion of tumors by enhancing mitochondrial reactive oxygen species (67). Conversely, the NMD components are often targeted for cancer treatment. Treatment with compound 11j, an SMG-1/SMG1 inhibitor, together with XR-2, an inhibitor of mouse double minute 2 homolog that degrades p53, upregulates the p53 pathway, preventing carcinogenesis (68). In addition, destabilization of SMG-2/UPF1 by inhibiting protein arginine methyltransferase 4, which methylates SMG-2/UPF1, leads to the generation of diverse antigens that sensitize cells to antiprogrammed cell death protein 1 treatment (69). Overall, these studies suggest the roles of the NMD pathway in cancer and potential therapeutic anticancer strategies targeting NMD.

## The role of NSD and NGD in aging and age-related diseases

The two RQC-associated mRNA surveillance mechanisms, NSD and NGD, play protective roles in cells against the accumulation of aberrant mRNAs and proteins. Recent studies suggest that NSD and NGD have crucial functions in aging and age-associated diseases. Ribosome-associated isolated 3′ UTRs are RNA fragments derived from mRNAs that have lost their coding sequences and 5′ UTRs but remain bound to ribosomes (70). Oxidative stress impairs the Fe–S cluster–containing ribosome recycling factor ABCE1/ABCE-1, causing ribosome stalling near stop codons and subsequent endonucleolytic cleavage *via* the NGD pathway. Such isolated 3′ UTRs accumulate in aged mouse and human brains, particularly in neurons with high oxidative stress, representing a molecular hallmark of impaired ribosome recycling during aging (70). In yeast, oxidative stress leads to the accumulation of oxidized mRNAs, containing 8-oxo-7,8-dihydroguanosine, a marker of oxidative RNA damage, which stalls translation and triggers degradation by the NGD pathway (71, 72). In addition, key components of NSD/NGD, PELO-1/DOM34/PELO, HBS-1/HBS1/HBS1L, and SKI7, a paralog of HBS-1/HBS1/HBS1L, are indispensable for oxidative stress tolerance, as they counteract the production of aberrant proteins (73). Moreover, the dysregulation of NSD and NGD processes contributes to protein aggregation, a hallmark of several age-associated diseases and aging (18, 74). mRNAs without stop codons, which are targets for degradation by NSD, can produce aberrant proteins associated with cellular defects, as exemplified by the self-aggregation of the nonstop variant REEP1 in human peripheral neuropathy patients (75). The protein products generated from mRNAs without stop codons are localized in the nucleoli and cause nucleolar deformation in *Drosophila* and cultured human cells (17). Such nucleolar deformation may impair ribosome biogenesis, leading to translational defects by releasing pre-60S subunits into the cytoplasm, thereby causing ribosome stalling (76). Impaired rRNA synthesis and reduced ribosome biogenesis also directly cause cellular senescence by activating p53 and the retinoblastoma protein, respectively (77, 78). Furthermore, a deficiency in NSD and NGD leads to widespread aggregation of endogenous proteins in yeast (74). These aggregated proteins are composed predominantly of highly abundant and actively translated proteins, supporting the idea that mRNA surveillance pathways prevent aberrant translation events that generate misfolded proteins and disrupt proteostasis. Thus, NSD and NGD collectively play pivotal roles in eliminating abnormal mRNAs that otherwise impair proteostasis, thereby preventing diverse age-associated diseases (Fig. 3*A*).

## The role of mRNA surveillance–associated RQC in aging and age-associated diseases

Ribosome stalling on mRNAs during translation can be caused by mRNA damage, changes in secondary structure, rare codons, or tRNA and amino acid deficiencies (Fig. 2*A*) (41, 72, 79, 80, 81). Ribosome stalling, followed by the collision of ribosomes, produces abnormal and dysfunctional nascent polypeptides that disrupt protein quality (Fig. 2*B*) (10, 11, 41, 82, 83, 84). Therefore, RQC, a cotranslational surveillance process, degrades nascent polypeptides and aberrant mRNAs, while recycling ribosomes and tRNAs, thereby contributing to the proper maintenance of protein quality (41, 82, 83, 84, 85, 86). Impaired RQC leads to an increase in abnormal proteins and their aggregation, which disrupts proteostasis, implying an intimate relationship between RQC and age-associated pathology (Fig. 3*B*) (1, 87).

In *C*. *elegans*, yeast, and killifish, ribosome stalling and collisions increase with age on polybasic stretches, decreasing the kinetics of translation elongation (10, 88). In addition, ribosome stalling leads to the accumulation of aberrant nascent peptides that are prone to aggregation with age. Ribosome stalling usually occurs on mRNAs with stalling-prone sequences, including those lacking a termination codon targeted by NSD or containing inhibitory structures that trigger NGD (89). The level and activity of NSD, NGD, and RQC components decrease with age (10, 11). In *C*. *elegans*, RQC/NSD and RQC/NGD reporter signals increase by nearly twofold in day 9 adults compared with day 1 adults, indicating a corresponding reduction in NSD, NGD, and RQC activity (11). The translation levels of key RQC components, including receptor for activated C kinase 1 (RACK-1/ASC1/RACK1), transcription factor 25 ribosome quality control complex subunit (K07A12.1/RQC1/TCF25), and Y82E9BR.18/RQC2/Clbn/NEMF, are reduced by over twofold during aging (10). These data suggest that decreased levels of RQC components lead to the impaired clearance of nascent polypeptides and accumulation of aggregated proteins. In addition, the mRNA levels of key NSD, NGD, and RQC components, such as *pelo-1/DOM34/PELO* and *skih-2/SKIV2L*, are reduced by approximately twofold during aging (11). Age-dependent impairment of mRNA surveillance and RQC exacerbates proteotoxic stress and disrupts proteostasis. This occurs by allowing ribosome stalling–derived aberrant nascent peptides to accumulate and by impairing autophagy through elevated mechanistic target of rapamycin (mTOR) signaling (39). Notably, PELO-1/DOM34/PELO and SKIH-2/SKIV2L, which are essential for resolving stalled ribosomes and degrading faulty mRNAs, are required for the longevity conferred by reduced insulin/IGF-1 signaling and for maintaining healthspan in *C*. *elegans*. Importantly, the role of PELO-1/DOM34/PELO is conserved in mammals, where its depletion in cultured human cells and mice accelerates cellular senescence, sarcopenia, and neurodegeneration (11). In the brain of the short-lived vertebrate African killifish, *Nothobranchius furzeri*, age-dependent increases in ribosome stalling on polybasic stretches lead to reduced translation of mRNAs enriched in basic amino acids (88). This translation reduction causes protein-transcript decoupling, in which mRNA levels remain unchanged, whereas protein levels decrease. The proteins undergoing the protein-transcript decoupling include ribosomal proteins, DNA and RNA polymerases, spliceosome components, and DNA repair factors, all of which affect aging. Together, these findings suggest that the age-dependent accumulation of ribosome stalling and the loss of NGD/NSD/RQC function contribute to organismal aging. In addition, ribosome stalling appears to disrupt mTOR/autophagy signaling, increasing protein aggregation and thereby exacerbating the organismal aging. However, the causal hierarchy between these processes remains to be clarified, and it is still debated whether mTOR activation stems directly from stalled ribosomes or arises secondarily from proteotoxic stress.

Impairment of RQC is mechanistically linked to diverse age-associated neurological disorders, including Parkinson’s disease (PD) and AD (Fig. 3*B*). Upon mitochondrial damage, stalled ribosomes on mitochondrial outer membrane–localized mRNAs recruit RQC factors, such as PELO-1/DOM34/PELO, ABCE-1/ABCE1, and the E3 ligase CCR4–NOT transcription complex subunit 4 (NTL-4/CNOT4), which ubiquitinates ABCE-1/ABCE1 to trigger phosphatase and tensin homolog–induced kinase 1 (PINK-1/Pink1/PINK1)–dependent mitophagy (90). Notably, the levels of ABCE-1/ABCE1 and HBS-1/HBS1/HBS1L are significantly decreased in the brains of PD patients. Furthermore, mitochondrial dysfunction impairs translation termination, leading to the nontemplated C-terminal extension of the mitochondrial outer membrane–associated complex-I 30 kD subunit (C-I30) (91). This extension is mediated by Y82E9BR.18/RQC2/Clbn/NEMF through the addition of alanine and threonine, a process known as CAT-tailing. These aberrant proteins with C-terminal extensions are incorporated into mitochondrial respiratory complexes or form cytosolic aggregates, disrupting oxidative phosphorylation and proteostasis. Enhancing RQC by overexpressing *eRF1* or *abce-1*/*ABCE1*, which encode key components of RQC, prevents the C-terminal extension of C-I30 and alleviates mitochondrial and neuromuscular defects in *Drosophila* PD models. Thus, RQC appears directly associated with mitochondrial dysfunction and PD. In the *Drosophila* AD model, which expresses the human amyloid precursor protein C-terminal fragment (APP.C99), ribosome stalling increases during the translation of APP.C99 (92). Dysfunctional RQC increases the aggregation of APP.C99, causing endolysosomal and autophagic defects (92). Enhancing RQC and eliminating aggregated APP.C99 alleviate neuromuscular degeneration and cognitive defects in the AD model, indicating that RQC function is crucial to the pathogenesis of AD (92). A recessive hypomorphic mutation in *Y54E10A*.*11/Ltn1/LTN1*, caused by an in-frame deletion, decreases age-dependent muscle function and increases dystrophic neurites and hyperphosphorylated tau protein in mice (45). Thus, Y54E10A.11/Ltn1/LTN1 regulates neurodegeneration pathology through RQC. Mutations in *Y82E9BR*.*18*/*RQC2*/*Clbn*/*NEMF*, the other key RQC factor gene that regulates CAT-tailing, cause neuromuscular degeneration in both mice and humans (93). The inhibition of translation initiation is one of the crucial steps in RQC for proper protein quality control. Mutations in *Grb10-Interacting GYF Protein 2* (*gyf-1*/*Gigyf2*/*GIGYF2*), which encodes a translation initiation repressor of RQC substrate mRNAs, cause motor dysfunction and neurodegeneration in mice (94). Furthermore, α-synuclein-positive neuritic plaques accumulate in the brainstem and cerebellum of *gyf-1/Gigyf2/GIGYF2* mutant mice (83). Thus, translational control at the initiation stage is critical for reducing translational errors and maintaining neuronal integrity. Collectively, these studies demonstrate that RQC actively contributes to maintaining protein quality and neuronal integrity during aging, rather than merely correlating with these processes (Fig. 3*B*).

## Concluding remarks

mRNA surveillance mechanisms, including NMD, NSD, and NGD, play crucial roles in maintaining cellular and organismal health by enhancing mRNA and protein quality. NMD eliminates transcripts with PTCs, whereas NSD and NGD degrade transcripts, causing ribosome stalling and collisions, which are resolved by RQC. These surveillance processes contribute to normal cellular function and play crucial roles in aging and age-associated diseases.

Disrupted RNA and protein quality control is prevalent in various genetic diseases. Approximately 11% of gene lesions that are known to be responsible for inherited human diseases are caused by nonsense mutations (95). These pathogenic nonsense mutations generate PTCs, resulting in the production of aberrant proteins. Activating the NMD pathway can target and eliminate PTC-containing mRNAs, thereby preventing the production of potentially pathogenic proteins. Transcriptome analysis showed that 26% (33/127) of the genes upregulated in NMD-deficient lymphocytes and 15% (8/54) of those upregulated in *smg-2*/*UPF1* knockdown cells overlap with genes upregulated in the cerebellum of ALS patients carrying the *C9orf72* repeat expansion (53). This overlap was statistically significant, indicating the strength of this relationship. In a *Drosophila* model, *C9orf72* repeats inhibit NMD by reducing the formation of processing bodies (P-bodies), leading to the accumulation of NMD substrates and neurodegenerative phenotypes (53). Although this study implicates decreased P-body abundance in NMD inhibition, P-bodies are dispensable for NMD in cultured human cells (96). Overexpression of *smg-2*/*UPF1* or *smg-3*/*UPF2* alleviates neurotoxicity and degenerative symptoms (53), supporting the idea that reactivation of the NMD pathway represents a potential therapeutic approach for ALS prior to neurodegeneration.

Emerging evidence suggests that NSD and NGD, along with RQC, have crucial roles in aging and the pathogenesis of age-associated diseases. However, studies rarely focus on clinical approaches in human diseases derived from RQC dysfunction. Recently, RQC dysfunction has been implicated in the pathogenesis of age-associated diseases, raising the possibility of enhancing RQC to treat diseases, such as AD, PD, and ALS. In particular, clinically activating the RQC pathway or its key components may prevent premature aging and delay the onset of age-dependent chronic diseases.

## Conflict of interest

The authors declare that they have no conflicts of interest with the contents of this article.
